# Barcoding Human Physical Activity to Assess Chronic Pain Conditions

**DOI:** 10.1371/journal.pone.0032239

**Published:** 2012-02-23

**Authors:** Anisoara Paraschiv-Ionescu, Christophe Perruchoud, Eric Buchser, Kamiar Aminian

**Affiliations:** 1 Laboratory of Movement Analysis and Measurement, Ecole Polytechnique Federale de Lausanne (EPFL), Lausanne, Switzerland; 2 Anesthesia and Pain Management Department, EHC, Hospital of Morges, Morges, Switzerland; 3 Department of Anesthesiology, University Hospital Center and University of Lausanne, Lausanne, Switzerland; National University of Ireland Maynooth, Ireland

## Abstract

**Background:**

Modern theories define chronic pain as a multidimensional experience – the result of complex interplay between physiological and psychological factors with significant impact on patients' physical, emotional and social functioning. The development of reliable assessment tools capable of capturing the multidimensional impact of chronic pain has challenged the medical community for decades. A number of validated tools are currently used in clinical practice however they all rely on self-reporting and are therefore inherently subjective. In this study we show that a comprehensive analysis of physical activity (PA) under real life conditions may capture behavioral aspects that may reflect physical and emotional functioning.

**Methodology:**

PA was monitored during five consecutive days in 60 chronic pain patients and 15 pain-free healthy subjects. To analyze the various aspects of pain-related activity behaviors we defined the concept of PA ‘barcoding’. The main idea was to combine different features of PA (type, intensity, duration) to define various PA states. The temporal sequence of different states was visualized as a ‘barcode’ which indicated that significant information about daily activity can be contained in the amount and variety of PA states, and in the temporal structure of sequence. This information was quantified using complementary measures such as structural complexity metrics (information and sample entropy, Lempel-Ziv complexity), time spent in PA states, and two composite scores, which integrate all measures. The reliability of these measures to characterize chronic pain conditions was assessed by comparing groups of subjects with clinically different pain intensity.

**Conclusion:**

The defined measures of PA showed good discriminative features. The results suggest that significant information about pain-related functional limitations is captured by the structural complexity of PA barcodes, which decreases when the intensity of pain increases. We conclude that a comprehensive analysis of daily-life PA can provide an objective appraisal of the intensity of pain.

## Introduction

Pain is one of the major universal experiences of human beings defined by the International Association for the Study of Pain (IASP) as ‘*an unpleasant sensory and emotional experience associated with actual or potential tissue damage, or described in terms of such damage’* [1]. The modern theories that led to the IASP definition of chronic pain are rooted in many centuries of human philosophical and scientific thinking. One of the most influential developments was the *gate control theory* developed in 1960s by Wall & Melzack [2]. This theory proposes the existence of a neurophysiologic gating mechanism in the brain and spinal cord that can be excited or inhibited by a variety of influences including sensory-discriminative, affective-emotional and cognitive-evaluative components. The key point emerging from this theory is that pain is not only a sensation but a multi-dimensional personal experience [3] .

Despite significant progress to understand pain mechanisms, the assessment of chronic pain in clinical practice remains a major challenge that involves multidimensional outcome domains such as *pain intensity*, *emotional functioning* and *physical functioning* [4]. A number of validated tools such as the visual analog scale and quality of life questionnaires have been developed to assess pain but they have several limitations [5]: *(i)* they relay on self-reporting and recall and are therefore subjective and prone to biases [6]; *(ii)* they cannot be used in subjects with cognitive impairment as well as in some of the elderly and/or very young patients [7] ; *(iii)* they fail to capture the dynamic nature of pain and its impact on the aspects of daily-life functioning since the assessment/quantification of pain status is based on a static index at one point in time. Individuals who suffer from chronic pain may differ from healthy pain-free subjects not only in how they feel but also in how they behave. An important aspect of pain behavior is reflected in the physical functioning/activity since pain sufferers may have an altered gait pattern, move slower and be more likely to interrupt or avoid painful activities [8], [9]. Unlike most of the self-reporting, inherently subjective pain outcomes, the monitoring of behavioral patterns of daily physical activity may provide an objective and dynamic integrated appraisal of the impact of pain on the physical, social and emotional functioning of chronic pain patients.

Physical activity (PA) has many dimensions that can be characterized and quantified, such as the *type of activity* (e.g. sitting, standing, walking, lying), its *duration*, its *intensity* (e.g. gait speed, motility), its *frequency* (e.g. number of postural changes, number of walking episodes) and its *patterns* i.e. *the temporal sequence/organization* of various activities. Under real-life conditions these parameters can be influenced by a disease but also by non-pathological factors such as age, working status, and response to unexpected events [10], [11], [12]. Basic quantitative PA metrics such as the daily walking distance, the speed, and the percent of time spent lying may be useful to assess the efficacy of a treatment when patients serve as their own control [9]. However, studies that used global quantitative PA metrics to assess differences between chronic pain patients and healthy subjects have been essentially negative. In other words averaged/global parameters such as the total daily walking distance or the number of steps may be similar or even equal in patients and healthy controls, as is, incidentally, the average metabolic expenditure measured by double labeled water [13], [14], [15]. Yet, significant differences have been found when the temporal organization of PA patterns was investigated in trials comparing healthy subjects to patients with various conditions such as pain syndromes, chronic fatigue, degenerative neurological diseases and advanced aging [16], [17], [18], [19].

When PA is monitored over long periods of time, parameters related to the different dimensions can be used to define *time-series* or *patterns* which may contain significant information hidden in their *temporal structure* [16]. Looking at a measure of PA in the context of time (temporal/dynamical structure) enables a better understanding of behavioral features related to the ability of the subject to adapt to internal states (pain, fatigue, mood, etc) and to respond to external/environmental demands. In chronic disease conditions it is postulated that various factors including neurological dysfunctions, pain, fear of movement, mood, and coping strategies reduce the variety and intensity of body movements and activities resulting in a decrease in the *complexity* of PA patterns. This is supported by previous studies that investigated complexity in terms of *long-term power-law (fractal) correlations* embedded in time series generated from *one specific PA parameter* such as forearm or ankle motion [17], [18], [19], posture allocation [16], walking activity [20] or gait [21], [22]. The results of these studies showed that power law, time-invariant dynamic patterns of fluctuations characterize PA in healthy conditions but this feature tends to fade away with aging [18] or disorders such as Alzheimer's disease [18], chronic pain syndrome [16], and chronic fatigue syndrome [19].

Based on the above hypothesis and background, the present study is a further step in the investigation of the dynamics of patterns (sequences) generated from a succession of various *PA states*. Each state represents a combination of *many dimensions of PA/body movement abilities* (e.g. type, duration and intensity) that occur in a defined time-window. The significant information embedded in these patterns - metaphorically named ‘barcodes’ - was found to be related to the *structural complexity*, which captured the variety of PA states as well as their occurrence in time. The *methodological* objective was to describe the practical issues related to the definition and the analysis of PA barcodes using complementary metrics, which capture most of the clinically meaningful information. The *clinical* objective was to evaluate the reliability of the defined metrics to discriminate between matched groups (age, employment status) of subjects with different *pain intensities*.

## Materials and Methods

### Subjects and study design

We performed a retrospective analysis on data that were collected prospectively in an observational longitudinal study designed to assess the PA in chronic pain patients treated with spinal cord stimulation (SCS). After approval of the ethical committee of the University of Lausanne, Switzerland, and written informed consent was obtained, 60 patients suffering from chronic pain caused by failed back surgery syndrome (n = 21), spinal stenosis (n = 19), peripheral vascular disease (n = 8), and combined pathologies (n = 12) were enrolled. All patients reported pain-related limitations of their walking perimeter and were candidates for SCS therapy. All patients were referred to the Pain Management Centre of the Hospital of Morges, Switzerland because of persistent pain despite optimal medical management. As the main inclusion criterion was the eligibility for SCS treatment, the group was not homogeneous in terms of pathologies and demographic characteristics. A group of 15 *pain free* healthy volunteers were enrolled in the protocol. These subjects were recruited from the patient's relatives or the medical staff of the clinic.

Pain was measured using a visual analogue scale (VAS) from 0 to 10. All subjects were asked to rate the *usual* pain intensity experienced during each day of PA measurement. VAS score modifications in excess of 30% are considered to reflect clinically significant difference in pain intensity [23]. Post-hoc subjects groups were defined according to the intensity of pain and matched for demographic characteristics.

The pain intensity was categorized as ‘*no pain*’, ‘*mild*’, *‘moderate*’ and ‘*severe*’ based on the VAS as follows: ‘no pain’: VAS = 0, ‘mild’: VAS = 1 to 3, ‘moderate’: VAS = 4 to 6, ‘severe’: VAS = 7 to 10 [5]. Table 1 shows the clinical and demographic data according to the partition of the subjects in four subgroups. To quantify the impact of clinically different pain levels on PA independently of demographic covariates such as age and employment status the subgroups were matched in two pairs and the comparison was performed as follows: *No Pain* vs. *Severe Pain* in the *Middle Age* groups and *Moderate Pain* vs. *Severe Pain* in the *Old Age* groups.

**Table 1:** Characteristics of each group (mean±SD) and statistical differences between groups.

	*No Pain, Middle Age (n = 15)*	*Severe Pain, Middle Age (n = 25)*	*Differences between groups*	*Moderate Pain, Old Age (n = 16)*	*Severe Pain, Old Age (n = 19)*	*Differences between groups*
Pain intensity (0 to 10)	0	7±1.3	*p* = 10^−21^	3.6±1.4	7.7±1.3	*p* = 3*10^−9^
Age (yr)	57±14	54±9	*p* = 0.63	71±14	74±8	*p* = 0.19
Gender, *n* males (%)	8(53%)	15(60%)	*p* = 0.32	9(56%)	10(52%)	*p* = 0.61
Height (m)	168±3	167±7	*p* = 0.62	168±5	168±8	*p* = 0.97
Weight (kg)	71±10	76±24	*p* = 0.47	72±13	76±12	*p* = 0.35
BMI (kg/m^2^)	24.6±2.5	26.7±7.2	*p* = 0.29	25.1±3.3	26.5±3.7	*p* = 0.25
Employed, n (%)	13(86%)	25(100%)	*p* = 0.06	2(0.13%)	0(0%)	*p* = 0.1
Diagnosis, n (type)	-	4(SS) 13(FBSS) 2(CRPS) 3(PVD) 1(LB) 1(PN) 1(HD)	-	6(SS) 4(FBSS) 3(PVD) 1(LB) 1(Meralgya) 1(DA)	9(SS) 4(FBSS) 2(PVD) 1(PN) 1(DA) 2(HD)	-

The effect of pain intensity on PA was compared on age-matched groups, i.e. *No Pain* vs. *Severe Pain* in the *Middle Age groups* and *Moderate Pain* vs. *Severe Pain* in the *Old Age groups.* It can be observed that in the *Middle Age* groups the mean difference in pain intensity is about 70% while it is only about 35% in the *Old Age* groups.

Diagnosis: *SS = spinal stenosis; FBSS = failed back surgery syndrome; CRPS = Complex regional pain syndrome; PVD = peripheral vascular disease; LB = low back and leg pain; PN = polyneuropathy; DA = deafferentation; HD = Herniated disc.*

### Instrumentation and measurement protocol

The monitoring of PA was performed under free living conditions using three miniaturized data-loggers (55×40×18 mm, 50 g) stuck to the skin with medical adhesive patches (Coloplast Systems, Denmark) and Velcro (Velcro®,USA). The data-loggers are custom designed from commercial inertial sensors (bi-axial accelerometers, ADXL202, ±2 g and uni-axial gyroscope, ADXRS300, 300°/sec), memory, electronics for data acquisition and rechargeable batteries. One device was fixed on the sternum to measure the trunk vertical and frontal accelerations, and the angular velocity in the sagittal plane. Two devices were fixed on one leg aligned with the medio-lateral axis of the thigh and shank, to measure vertical and frontal accelerations and the angular velocity of thigh and shank in the sagittal plane [24]. Body accelerations and angular velocities were synchronously recorded at a sampling rate of 40 Hz during five consecutive weekdays, continuously eight hours per day. The chronic pain patients were monitored before SCS treatment. All subjects were instructed to install the monitoring devices and start the recording in the morning before engaging in daily activities. In order to avoid skin sensitivities due to adhesives patches, it was allowed when started each monitoring day to slightly change the location of the device on the sternum and along the medio-lateral axis of thigh and shank. Since the biomechanical signals were recorded on rigid body segments the raw data and the performances of PA analysis algorithms were not affected.

### Defining the PA barcode

The basic idea of PA barcoding was to combine different PA dimensions in order to define PA states. A numerical symbol was assigned to each PA states so that the motor activity behavior during the observation period appeared encoded in a sequence of symbols. The sequence was then analyzed to provide PA metrics and was represented as a color barcode to provide global illustrative visual information.

#### Quantifying PA dimensions

We estimated the *type of activity* and its *duration* in terms of posture allocation periods, i.e. sitting (*Si*), lying (*Ly*), standing (*St*), and walking (*Wk*) using the inertial data recorded on the trunk, the thigh and the shank [24]. The *intensity* of each activity was estimated from: *(1)* the mean walking cadence (*cad*) during each detected walking period, and *(2)* the mean value of the trunk acceleration norm (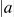) estimated on successive time-windows during periods of *Si/Ly* and *St*.

According to the algorithms developed previously [24] the parameters related to the different PA dimensions were estimated using five kinematics signals from the total of nine that were recorded by the three data-loggers. Similar parameters can be estimated using an even more reduced set of signals (trunk vertical and frontal accelerations and thigh frontal acceleration) but with decreased accuracy for walking detection especially in pathological/elderly conditions [24].

#### Encoding PA dimensions into PA states

The parameters related to the type, the intensity, and the duration were combined within successive time-windows of one-second duration to define the various PA states. A numerical symbol was assigned to each PA states using the encoding procedure illustrated in Table 2:

1. if the type of PA was identified as *Ly/Si* and the intensity of trunk acceleration was below or above a specific threshold then two PA states were defined and encoded with *‘1’* and *‘2’*, respectively;
2. if the type of PA was identified as *St* and the intensity of trunk acceleration was within four specific ranges then four PA states were defined and encoded with *‘3’, ‘4’, ‘5’, ‘6’*;
  The choice of thresholds used for trunk acceleration norm i.e. *th_a1_* = 0.2, *th_a2_* = 0.4, *th_a3_* = 0.6 (g) was based on typical values of trunk acceleration during postural transitions (*Si-St/St-Si*) and usual homework activities (mild, moderate and intense, respectively) [25], [26], [27].
3. if the type of PA was identified as *Wk* and the mean walking cadence was within four specific ranges and the *duration* of the walking episode was within three specific ranges then twelve possible PA states were defined and encoded with numbers from *‘7’* to *‘18’*.
  The thresholds used for walking cadence i.e. *th_c1_* = 50, *th_c2_* = 80, *th_c3_* = 140 (steps/min) and duration of continuous walking episodes, *d*, i.e. *th_d1_* = 30, *th_d2_* = 120 (s) were chosen based on typical values of cadence during slow, medium and fast walking and the distribution of the duration of the walking episodes that are characteristic for indoor and outdoor activity.

**Figure 1:**
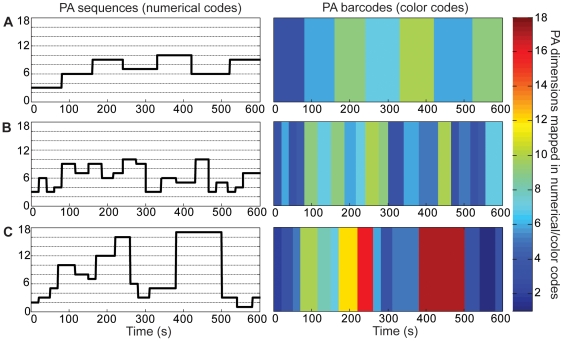
Examples of PA patterns represented as symbolic/numerical sequences (left panel) or color barcodes (right panel) : (A) and (B) have a similar distribution of states but differ in their sequential structure. The pattern shown in (C) differs from (A) and (B) by both, the distribution/variety of states and their sequential structure.

This encoding procedure provided the representation of the patterns of PA as successions of 18 possible states. Mathematically, such representation corresponds to symbolic sequences over the alphabet *Ω_18_* = [*1,2,3,….,18*] of length *α* = 18 that can be visualized as color barcodes. Figure 1 emphasizes that PA patterns covering similar time spans may appear different in many aspects such as the *number*, the *variety* and the *succession* of states. Each of these aspects contributes to an aggregate property of such patterns that is called ‘*complexity*’.

**Table 2:** Mapping physical activity dimensions into physical activity states (*PAS*): the various physical activity (PA) dimensions i.e. the type (*Sitting/Lying→Ly/Si, Standing→St, Walking→Wk*), intensity (trunk acceleration norm*→*I*a*I, walking cadence*→cad*) and duration (*d*) are combined to generate PA states (*PAS*) that are encoded into numerical symbols using the alphabet *Ω*_18_ = [1,2,3,…,18] of length α = 18.

*PA type*	*PA intensity*	*PA duration*	*PAS encoded in numerical symbols*
*Ly/Si*	|*a*|≤*th_a_*_1_		‘1’
*Ly/Si*	|*a*|>*th_a_*_1_		‘2’
*St*	|*a*|≤*th_a_*_1_		‘3’
*St*	*th_a_*_1_<|*a*|≤*th_a_*_2_		‘4’
*St*	*th_a_*_2_<|*a*|≤*th_a_*_3_		‘5’
*St*	|*a*|>*th_a_*_3_		‘6’
*Wk*	*cad*≤*th_c_*_1_	*d*≤*th_d_*_1_	‘7’
*Wk*	*th_c_*_1_<*cad*≤*th_c_*_2_	*d*≤*th_d_*_1_	‘8’
*Wk*	*th_c_*_2_<*cad*≤*th_c_*_3_	*d*≤*th_d_*_1_	‘9’
*Wk*	*cad*>*th_c_*_3_	*d*≤*th_d_*_1_	‘10’
*Wk*	*cad*≤*th_c_*_1_	*th_d_*_1_<*d*≤*th_d_*_2_	‘11’
*Wk*	*th_c_*_1_<*cad*≤*th_c_*_2_	*th_d_*_1_<*d*≤*th_d_*_2_	‘12’
*Wk*	*th_c_*_2_<*cad*≤*th_c_*_3_	*th_d_*_1_<*d*≤*th_d_*_2_	‘13’
*Wk*	*cad*>*th_c_*_3_	*th_d_*_1_<*d*≤*th_d_*_2_	‘14’
*Wk*	*cad*≤*th_c_*_1_	*d*>*th_d_*_2_	‘15’
*Wk*	*th_c_*_1_<*cad*≤*th_c_*_2_	*d*>*th_d_*_2_	‘16’
*Wk*	*th_c_*_2_<*cad*≤*th_c_*_3_	*d*>*th_d_*_2_	‘17’
*Wk*	*cad*>*th_c_*_3_	*d*>*th_d_*_2_	‘18’

### Analyzing the PA barcode

The PA symbolic sequences/barcodes to be analyzed were obtained by concatenation of data from the five recording days. Each data point/sample of the PA barcode represents a PA state calculated for time-windows of one second, so that the length of the analyzed barcodes (*N* = 5 days×8 hours×3600 s = 144.000) was sufficiently long to have a robust estimation of complexity metrics [28].

#### Complexity metrics

The choice of a measure of complexity must be based on its ability to reveal clinically relevant features of movement behavior and to discriminate between experimental groups. The illustrative examples in Fig. 1 suggest that meaningful information resides in the variety, the temporal dynamics, and the duration of PA states. These differences can be quantified using *structural complexity measures* defined as *structural-static* and *structural-dynamic* [28], [29]. Together, these features may quantitatively characterize the complexity of PA symbolic sequences/barcodes which can be observed both, in the structure (variety/amount of different states) and the temporal behavior (ordering of different states).

Structural-static measures allow the quantification of the amount of different PA states while structural-dynamic measures permit the quantification of the amount of different states in the sequence and a description of transitions/succession between states. Structural-dynamic measures are said to be ‘sequence-sensitive’ because their values depend on the order of PA states in the sequence [29].

We used three complementary complexity measures to quantify the information embedded in the PA barcodes, and to investigate the effectiveness of the proposed methodology to differentiate between chronic pain conditions (intensities): the *information entropy*, *the Lempel-Ziv complexity*, and *the sample entropy*.

*Information Entropy (H)* is defined as *structural-static complexity metric* i.e. a measure of variety of PA states in the barcode, that takes a large (small) value if there are many (few) kind of PA states in the barcode. The information entropy is calculated as 
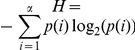
 where *α* is maximal number of defined states in the alphabet (*α* = 18) and *p*(*i*) denotes the probability of *i*-th symbol. The normalized information entropy is defined as 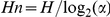 and varies between 0 and 1 inclusive [30].

*Information entropy* is sensitive to the diversity of PA states in the sequence/barcode but is insensitive to the dynamical structure of the sequence/barcode, i.e. the temporal ordering of PA states. This can be illustrated by considering the two sequences: 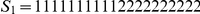 and 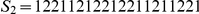; both have the same number of symbols/states (*α* = 2) therefore the same entropy, however their temporal structure is different. The difference in the dynamic structure can be quantified using sequence-sensitive complexity measures, i.e. complexity measures which change when the order of the symbols is changed.

*Lempel-Ziv complexity (LZC)* is a *structural-dynamic*, non-parametric measure that captures the number of ‘new’ sub-patterns discovered as the sequence evolves from left to right. The *LZC* is closely related to *Kolmogorov complexity* which is the central concept of complexity analysis of symbolic sequences [31]. According to this concept, the complexity/information content carried in a symbolic sequence is given by the length of the shortest algorithm (computer program) that can reproduce the sequence. Since the shortness of such algorithm cannot be computable, several alternative calculations of complexity have been proposed in literature. Lempel and Ziv explored a different approach to the problem of complexity of a specific symbolic sequence [32]. They linked the notion of complexity to the generation rate of new sub-patterns along a sequence *S*(*N*, *α)*, of length *N* with symbols from an alphabet of size *α* and proposed a useful measure defined as 
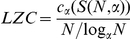
, where 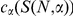 is the number of sub-patterns in the decomposition of 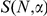. This measure can be efficiently calculated with the algorithm provided by Kaspar and Schuster [33], [34]. This algorithm is based on an iterative procedure of identification of sub-patterns in a symbolic sequence, 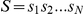, as follows. Suppose that *S* has been reconstructed by the program up to the symbol 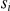 and that the sequence *S* up to 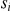 contains two sub-patterns i.e. 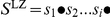 where the dot (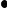) indicates a new detected sub-pattern (the first symbol in the sequence being always the first ‘sub-pattern’), and 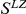 denotes the Lempel-Ziv processed sequence *S*. Let us check whether the rest of the sequence *S*, i.e. 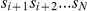 contains new sub-patterns. First, it is tested if 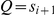 is contained in the vocabulary of 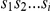; if 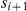 is a symbol in 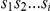 it is checked if 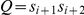 is contained in the vocabulary of 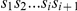 and so until the new combination *Q* is not found in the vocabulary of the sequence already processed (vocabulary being defined as symbols or succession of symbols in the sequence S). In this case the combination *Q* is inserted as a new sub-pattern and the number 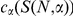 is incremented.

As a simple illustrative example, when applying the algorithm described in [34] to the two sequences:


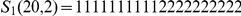
 and 
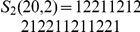
 we obtain after computation:


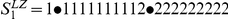
 and 
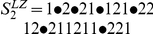
, equivalently with the complexity 
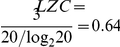
 for S*_1_* and 
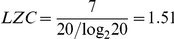
 for S*_2_*. A larger *LZC* implies a greater chance of the occurrence of new sub-patterns in the sequence and, thus, a more complex temporal/dynamical behavior.

The examples above illustrate that the entropy *H (Hn)* is concerned only with the expected occurrence of each symbol in the sequence and not with the temporal structure (ordering) of the sequence (since it is defined per symbol and not as a block entropy). The *LZC* relies on the concept of (block) entropy to quantify the information in the sequence, but only after analyzing the temporal structure of the sequence [35]. In this way, the *LZC* quantifies the notion of complexity in the Kolmogorov sense as well as in the statistical (entropic) sense.

*Sample entropy* (*SampEn*) is a *structural-dynamic*, parametric measure that quantifies the regularity of a symbolic sequence (time series) by analyzing the presence of similar sub-patterns in the data sequence. It is defined as the negative natural logarithm of the conditional probability that two sub-patterns similar for *m* points remains similar at the next point (*m*+1). In order to compute the *SampEn*, the symbolic sequence *s*(*n*), 1≤*n* ≤*N* is divided into overlapping subsequences of size *m*, defined as *y*(*i*) = [*s*(*i*), *s*(*i*+1),…,s(*i*+*m*-1)], 1≤*i*≤*N*-*m*+1. The probability that two sub-patterns match for *m* points, 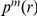, is computed by counting the average number of sub-pattern pairs for which the Euclidian distance is lower than a tolerance *r*. Similarly, 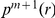 is defined for (*m+1*) points and *SampEn* is then calculated as:
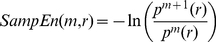
(1)It provides a non-negative finite index, where high values suggest high complexity, irregularity and unpredictability in the symbolic sequence. The tolerance *r* has typical values between 10 and 25% of the standard deviation of the symbolic sequence [36].

Although both, *LZC* and *SampE* estimate the regularity in PA sequences their computational approach is totally different, thus providing two distinct aspects of the dynamic complexity embedded in the sequences. The *SampEn* provides a measure of how regular (similar) consecutive PA states are generated one after the other in time, according to a type of analysis that takes place step-by-step. The *LZC* method uses sub-patterns of increasing length of consecutive states and can identify different sub-patterns of successive states, providing a statistical evaluation of occurrence and recurrence of these sub-patterns along the entire PA sequence. Because of their ability to detect and quantitatively characterize structures that are invisible to distribution-based measures like information entropy, dynamic complexity measures such as Lempel-Ziv complexity and Sample entropy are becoming important complementary metrics in studies of behavior [37], [29].

#### Quantitative/global metrics

The ‘traditional’ assessment of PA and functional capacity of subjects is expressed in terms of the time spent walking and/or standing (e.g. estimated globally as % over the monitoring time) [9], [38], [39]. In order to investigate the effectiveness of the new complexity metrics, comparison with the traditional assessment was investigated. We defined a quantitative metric as the % of time spent in ‘active’ PA states, i.e. PAS = 3 to 18 (denoted *%activity*).

#### Composite scores

When several metrics quantifying various specific aspects of PA are available, it is possible to combine them into a single parameter that may increase the ability of PA barcode to discriminate patients with high versus low chronic pain intensity. We studied and compared two approaches: *(1)* a *deterministic score* based on an ad-hoc combination of the different metrics and *(2)* a *statistical score* based on a statistical dimensionality reduction method.

The *composite deterministic score* (*CDS*) was defined as:
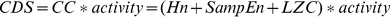
(2)where *CC* was defined as the *composite complexity*:
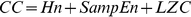
(3)It is noted from eq. (2) that the *CDS* was defined as the product between the composite complexity, *CC*, and the % *activity*. This definition could be justified as follows: *(1)* the complexity measures are all normalized (range 0 to approx. 1) therefore the appropriate mathematical operator to integrate their values into the composite complexity index, *CC*, was the addition; *(2)* the %*activity* theoretically ranges from 0 to 100, therefore the most appropriate operator to combine it with *CC* was the multiplication.

The *composite statistical score (CSS)* was defined using the linear discrimination analysis (LDA) method. LDA is a classical statistical approach for supervised dimensionality reduction and classification [40]. It focuses on the association between multiple (normally distributed) independent variables and a categorical dependent variable by forming a composite of weighted independent variables. One possible advantage of this methods compared to *CDS* is to determine the extent of any of the composite variables to discriminate between two (or more) pre-existing groups. Specifically, in our application the independent variables are the subjects' PA metrics (verified for positive Normality test) arranged in a set **X** = **[x^(1)^, x^(2)^,…,x^(N)^]** ***^T^***, with **x** = [*Hn, SampEn, LZC, activity*], *N*1 of which belong to a pain intensity class ω_1_ and *N2* to a pain intensity class ω_2_ (i.e. ω_1_ = ‘*no pain*’ and ω_2_ = ‘*severe*’ or ω_1_ = ‘*moderate* and ω_2_ = ‘*severe*’; *N* = *N*1+*N*2 subjects, n = 2 classes). The *composite statistical (discriminative) scores* corresponding to each subject and each class are calculated with the [*N*x*n*] matrix **CSS**, defined as:
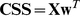
(4)where **w** *^T^* is a [*m*x*n*] weight matrix (*m* = 4 independent variables/PA metrics) with the weights *w_ij_* obtained to maximize the separability between the two classes (ω_1,_ω_2_) through the four metrics. Weights with large absolute values reflect greater discriminating ability to their corresponding variables.

### Statistical analysis

The impact of clinically different pain intensities on PA was compared between age-matched groups, i.e. *No Pain* vs. *Severe Pain* in the *Middle Age groups* and *Moderate* vs. *Severe Pain* in the *Old Age groups*, as reported in Table 1. The parameters characterizing the PA sequences/barcodes were estimated for each subject then the mean and standard deviation values were calculated for each group of subjects. The distribution of parameters in each group was tested for Normality using Shapiro-Wilk test. Based on the Normality test the differences between groups were assessed using two-sided Student's t-test or the nonparametric Mann-Whitney test. For all parameters, we calculated Cohen's *d* to determine effect size and percentage of non-overlap between groups [41]. Correlations between parameters were quantified using Spearman rank-correlation test.

The ability of the composite PA scores (CDS and CSS) to differentiate between the groups of subjects was quantified and compared using the receiver operator characteristic (ROC) curve and the area under the curve (AUC). The AUC is a summary measure of differentiation accuracy, lying in the range (0.5, 1), with 1 indicating perfect discrimination and 0.5 indicating no discrimination capacity [42]. The significance level was set at p<0.05 for all comparisons.

## Results

### PA barcode representation

**Figure 2:**
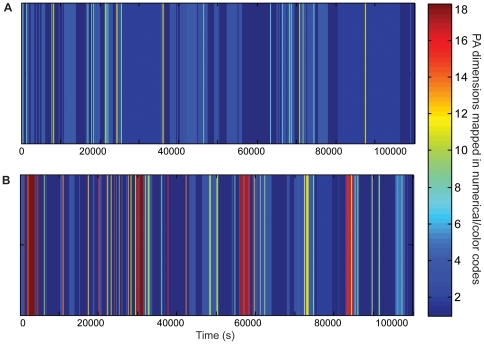
Examples of PA barcodes recorded in two aged-matched subjects: a chronic pain patient (A) and a healthy pain free subject (B): the two barcodes differ in both, the variety of PA states and their temporal distribution. The suggestion is that the chronic pain patient was not able to dynamically alternate between various body movements/activities, probably because of pain intensity and/or other factors such as fear of movement and activity avoidance.

Figure 2 illustrates an example of PA barcodes recorded from a chronic pain patient (Fig. 2A) and a healthy subject (Fig. 2B). It can be observed that compared to the healthy subject, the barcode of the chronic pain patient appears poor in high/intense activity states (high numbers/warm colors) suggesting an inability to perform intense movements such as long continuous walking, and to dynamically alternate various body movements.

### Discriminative features of PA metrics

#### Individual metrics

**Figure 3:**
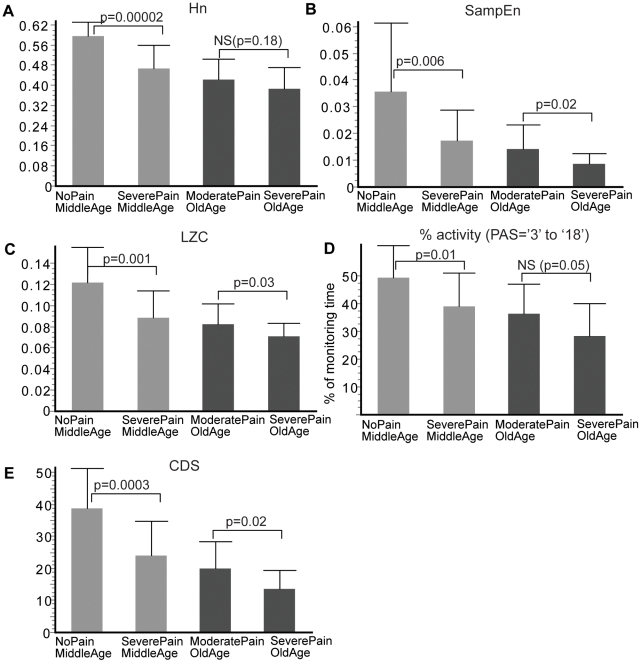
Metrics characterizing PA barcode (mean±SD) : structural-static complexity quantified by normalized *information entropy (Hn)*, (A); structural-dynamic complexity quantified by *Sample entropy* (*SampEn*) and *Lempel-Ziv complexity (LZC)*, (B), (C); classical PA metric quantifying the percent of time spent in *activity* (walking and standing, i.e. *PAS* = 3 to 18) (D); composite deterministic score (*CDS*) which integrates all defined metrics (E).

The analysis showed that all defined PA metrics decreased when pain intensity increased as illustrated in Fig. 3 (mean±SD). The information entropy *Hn* (Fig. 3A) showed very significant differences between the *Middle Age* groups with about 74% non-overlap. The same trend was observed between the *Old Age* groups, but the differences were not statistically significant (28% non-overlap).

The sample entropy *SampEn* (calculated with *m* = 3, *r* = 1) and *LZC* (Figs. 3B, C) discriminated significantly between all groups. Effect size calculations for *SampEn* indicated non-overlap percentages of 56% in the *Middle Age* groups, and 49% in the *Old Age* groups. Similar results were obtained for *LZC*, with 63% non-overlap in the *Middle Age* groups, and 48% in the *Old Age* groups.

The %*activity* (Fig. 3D) decreased also with the intensity of pain but compared to the complexity metrics, the discrimination between groups was diminished. Effect size calculations indicated 50% and 43% non-overlap in the *Middle Age* groups and the *Old Age* groups, respectively.

#### Composite scores

**Figure 4:**
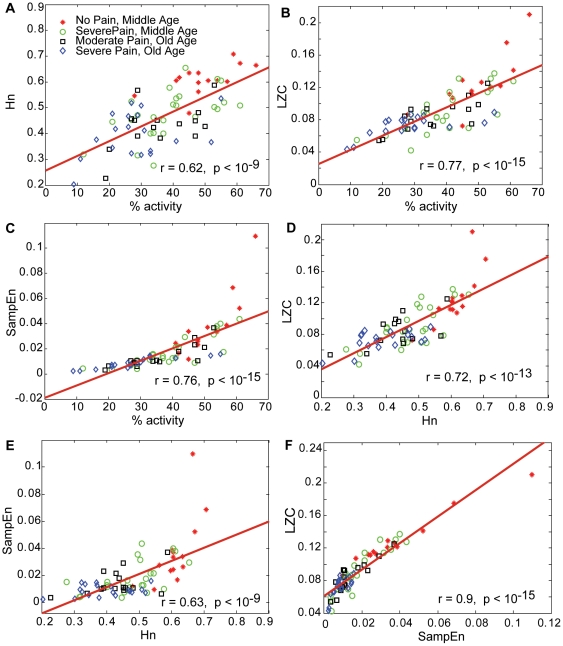
Correlations between metrics characterizing PA barcode.

The correlations illustrated in Fig. 4A–F indicate a degree of complementarities between the different metrics and justified our attempt to combine them into one score. Figure 3E shows the values of the composite deterministic score, *CDS*, which better captured the differences between groups. Effect size calculations indicated 67% non-overlap in the *Middle Age* groups, and 52% in the *Old Age* groups.

**Figure 5:**
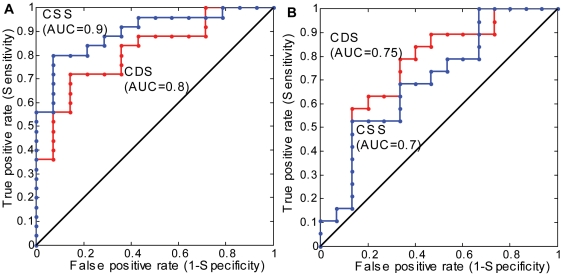
Receiver operator characteristics (ROC) curves and area under the curve (AUC) for the composite deterministic score (*CDS*) and the composite statistical score (*CSS*): *No Pain, vs. Severe Pain in the Middle Age groups* (A) and *Moderate Pain vs. Severe Pain in the Old Age groups* (B).

Figure 5 shows the comparative discrimination performances of the composite scores, *CDS* and *CSS* using the ROC curves and AUC. As illustrated in Fig. 5A, both scores separate well in the *Middle Age* groups with *CSS* indicating a relatively better performance (AUC = 0.9) than *CDS* (AUC = 0.8). As expected from the performances of individual metrics, the discrimination features of *CDS* (AUC = 0.75) and *CSS* (AUC = 0.7) were fair and quite similar in the *Old Age* groups.

## Discussion

This study suggests that specific patterns of PA (‘*barcodes’*) can be defined to provide information on pain-intensity related changes of the daily PA of a patient. It also confirms the hypothesis that chronic pain results in reduced complexity of PA patterns. The results are promising since they may provide the only objective assessment of the impact of chronic pain conditions, a topic of great importance in both clinical research and clinical practice.

### Methodological considerations

The essential feature of the barcode concept is to ‘*carry’* information that can be used for ‘*identification’*. In this context, the aim of the presented methodological approach was to define PA barcodes that carry information integrated at two levels: at a 1^st^ level by combining the different PA dimensions (type, intensity, duration) into PA states and at a 2^nd^ level by combining the metrics that characterize the succession of PA states (barcode) into a composite score. The ability of defined metrics to identify groups of patients with different pain intensities depends on the effectiveness to capture most of the clinically relevant information.

**Figure 6:**
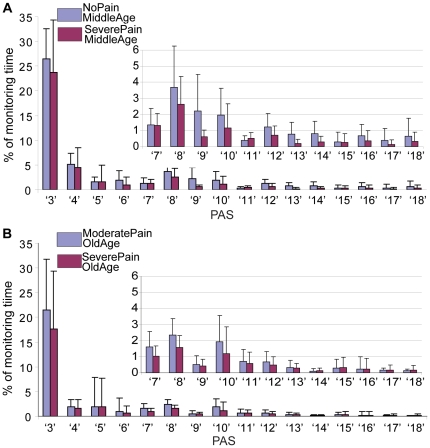
Quantitative assessment of intense physical activity states, *PAS* (mean±SD) : *No Pain vs. Severe Pain in the Middle Age groups* (A) and *Moderate Pain vs. Severe Pain in the Old Age groups* (B). These results indicated that elderly with either low pain or high pain levels are not able to perform very intense activities.

The information entropy, *Hn*, as a measure of structural-static complexity of PA barcode increased with the variety and statistical distribution of PA states. The results indicate that *Hn* discriminated significantly better than the other metrics (% *activity, SampEn, LZC*) in the *Middle Age* groups but not in the *Old Age* groups. This can be explained by the fact that in older subjects, the variety of PA states and in particular the capacity to perform high intensity activities is limited, primarily by age. This observation is supported by other studies that have reported a decrease in the duration and intensity of PA with age, especially in older women [11], [43]. The suggestion is that in older subjects, the assessment of physical functioning in terms of movement intensity does not carry additional information about the disease status, presumably because elderly patients are unable to perform very intense activities, regardless of whether they have pain or not as indicated by the percent of various PA states in Fig. 6A, B. This observation was supported also by the analysis of the cardinality of barcodes (i.e number of different types of PA states) which decreased when pain increased but not significantly.

The sample entropy, *SampEn*, and Lempel-Ziv complexity, *LZC*, as measures of structural-dynamical complexity of PA barcodes increased with the amount of different PA states and their temporal variability. These measures were more effective than the *% activity* and *Hn* to significantly discriminate low and high pain intensity in the *Middle Age* groups as well as in the *Old Age* groups. This suggests that the intensity of pain (and presumably pain conditions in general) may affect primarily the time-dependent dynamical behavior of body movements/activities rather than the cumulated time in active PA states (% *activity*) or the distribution/variety of PA states (*Hn*).

The composite scores *CDS* and *CSS* that integrate both global and temporal aspects of movement/activity behavior encoded in PA barcode appear to be the most effective discriminative measures. The statistical definition of *CSS*, based on weighted summation of PA metrics outperformed the ad-hoc deterministic definition of *CDS* based on mathematical considerations about the maximal range of PA metrics. This is explained by the fact that in the statistical model the weights are estimated to maximize the separation between groups. The deterministic definition might however be an alternative solution for studies with critical sample size for which a robust statistical model cannot be defined.

An important observation from the results presented in Fig. 3 is the influence of age (and implicitly employment status) on daily PA; similar pain levels seem to have different impact on patients with different ages (e.g. *Severe Pain*, *Middle Age* vs. *Severe Pain*, *Old Age*). This observation supports previous studies that showed a decrease in the amount [10] and the complexity of PA patterns with aging [18], [20] and highlights the importance to control for confounding factors by appropriate matching in studies looking at the impact of a disease on the daily PA.

### Clinical significance

There is growing evidence that chronic pain is associated with physical and psychological impairments that results in muscular disuse, anxiety and decreased quality of life [9], [14], [15], [38], [39], [44], [45]. The overall information contained in the PA barcode may be an expression of the freedom of movement and action and an indirect measure of health-related quality of life. The decrease in the complexity of PA barcodes with high levels of pain can be attributed to a loss of behavioral adaptability in daily living situations – a consequence of activity avoidance, fear of movement and possibly pain-related emotional factors (e.g. depression, anxiety). *Complexity* is a topic of increasing interest in modern physiology and it has been suggested that in biological systems, a decreased complexity is associated with reduced information content and decreased adaptability to an ever-changing environment [46], [47], [48], [49], [50]. A decreased complexity of the motor system, exemplified by the reduced range of movements or altered gait dynamics has been reported in several studies looking at age-related diseases, such as arthritis, stroke, Parkinson's disease [51], [52], [53], and other degenerative neurological diseases [21], [22]. In Parkinson's disease for example, the range of motor responses upon encountering a sudden obstacle is reduced, making it difficult for the patient to initiate and maintain useful movements [52].

Recent results in *experimental* pain research show that psychological factors such as pain-related fear and anxiety are associated with avoidance of physical activities resulting in poor PA performances and abnormal behavior. Significant correlations were found between pain and movement features such as amplitude/range of motion, acceleration/velocity and movement variability, defined as movement-to-movement change in motion pattern [44], [45]. A decrease in the overall movement amplitude was thought to reflect protective strategies that presumably minimize the impact of strain on painful muscles and joints. The reduced variability of movement is highlighted by behaviors that are characterized by movement that are much more stereotypical. This less complex pattern is thought to result from the deliberate or unconscious selection of movement patterns that produce the less pain [8], [9], [54], [55], [56].

A key issue in pain behavioral research is whether (and how) pain and pain-related fear affect the activities of *daily life*. Several studies investigated the relationship between pain-related escape/avoidance and disability levels in daily using self-reported assessments [56]. Although these studies indicated that pain-related fears affect the functioning in the daily life (including activities at workplace), the results were inherently subjective and more qualitative than quantitative. The results of the present study suggest that the analysis of PA barcode may have practical applications as a tool for the objective assessment of daily-life pain-related PA and behavior. This is important since the protective behavior associated with chronic pain (i.e. decreased movement duration, speed/intensity, and variability/complexity), can cause a number of other complications if it persists. The reliable assessment of daily functioning may help to understand the patient's pain condition and initiate a personalized pain management.

### Clinical limitations

There are several potential limitations regarding the interpretation of the present findings. A first limitation is that the relatively small sample size in each group may have led to under-powered statistical comparisons. A second limitation is that the retrospective cross-sectional nature of the study precluded a perfect matching between groups. While the groups were matched by age and occupational status (working or retired), they were inhomogeneous in terms of individuals' occupation type (profession) and pain mechanism or diagnosis. However as all patients had a pain-related limitation in their walking perimeter, the significance of the results is not expected to be affected. Finally, a more generic limitation is that for neither the “traditional” nor for the newly developed metrics there is (yet) an agreed definition of normal values and normal range. Similarly the clinical significance of the modifications that are observed remains to be established. Larger prospective and controlled studies are therefore needed to define normal PA, using sophisticated complexity metrics, which are needed to properly characterize chronic pain conditions whether in terms of the intensity of pain or possibly in terms of features that are disease-specific.

### Clinical perspectives

Pain has long been regarded as a diagnostic feature. The classical semiology of urethral colitis due to renal stone teaches that patients suffering from renal colic are “frantically” restless which is very different from patients with peritonitis who remain as immobile as possible to avoid pain. Similarly, patients with painful lower extremity neuropathy tend to move around as much as they can, while patients with hip arthritis tend to remain in the same position and avoid walking, which would increase pain. Hence pain does affect behavior (and PA) in a predictive way, irrespective of the intensity of the symptom.

Yet the clinical appraisal of behavioral patterns is crude and the traditional metrics are not contributive. The use of PA metrics that precisely and completely characterize the features of various chronic pain disorders may substantially improve our current assessment in a number of ways. Since it appears that the *mechanism of pain* is related to pain behavior, the reliable “barcoding” of PA may significantly improve the assessment of intricate pain conditions where the pain has more than one etiology.

Another potential useful application of PA barcoding is the assessment of patients who have communication difficulties such as the elderly or the cognitively impaired [57], [58]. Furthermore, the methodology could also be used in the follow-up of the functional status of various conditions such as age-related frailty, depression, post-stroke rehabilitation, neuromuscular diseases or heart failure. The ‘barcoding’ concept offers a flexible approach since it allows the definition of a large variety of PA states tailored to the clinical aspect of interest (movement behavioral features, sensor configuration for monitoring).
